# The Evolution of Fibromyalgia, Its Concepts, and Criteria

**DOI:** 10.7759/cureus.20010

**Published:** 2021-11-29

**Authors:** Frederick Wolfe, Johannes J Rasker

**Affiliations:** 1 Research, National Data Bank for Rheumatic Diseases, Wichita, USA; 2 Internal Medicine, University of Kansas School of Medicine Wichita, Wichita, USA; 3 Faculty of Behavioural Management and Social Sciences, Department of Psychology Health and Technology, University of Twente, Enchede, NLD

**Keywords:** misdiagnosis, somatic syndromes, diagnosis, fibromyalgia criteria, fibromyalgia

## Abstract

Fibromyalgia developed in the 1950s from a substrate of difficult to explain regional and widespread pain mixed with symptoms of psychosocial distress. Controversies regarding psychological issues were common. Multiple criteria arose to define the disorder, but each identified a different set of patients. The identification of widespread pain as a criterion changed the nature of the disorder by effectively eliminating regional pain as a component condition. The easy-to-measure and relatively reliable widespread pain criterion then came to define the disorder. In the primary care community, diagnostic criteria were largely ignored, and a substantial fraction of diagnosed patients with lower pain scores, particularly women and those with high non-pain symptom scores, were diagnosed. Non-pain symptoms were added back to the fibromyalgia definition and criteria in 2010. Recognition grew that fibromyalgia fit the description of a functional somatic disorder. The idea of fibromyalgia as a primary pain disorder with a neurobiological basis contended with fibromyalgia as a broader biopsychosocial disorder. It is increasingly recognized that fibromyalgia represents a dimensional, non-binary condition and that features of fibromyalgia extend to persons who do not satisfy the criteria. Severity assessments are now available but rarely used. The course of fibromyalgia is not well studied, and improvement and remission criteria have not been successfully defined. The future of fibromyalgia as a discrete disorder remains uncertain as features of fibromyalgia are increasingly observed in patients with multiple different medical conditions.

## Introduction and background

In this review, we trace the development of the idea of fibromyalgia and its criteria from the start of the disorder to the present, addressing some of the controversies associated with fibromyalgia and its criteria. In addition, we enumerate and address major problems with the idea and criteria of fibromyalgia. We use original datasets from three published sources to provide actual examples related to major fibromyalgia issues: An epidemiology study of the German general population [1], a US study of primary care patients [2], and analyses of patients from the US National Data Bank for Rheumatic Diseases (NDB) [3].

## Review

I know it when I see it

In 1964, US Supreme Court Justice Potter Stewart, “in what would become one of the most famous phrases in the entire history of the Supreme Court” wrote of pornography that perhaps he could never succeed intelligibly in defining it, “but I know it when I see it” [4]. The “I know it when I see it” idea also characterized early physician authors writing about fibromyalgia as they could not “intelligibly” define fibromyalgia but knew it when they saw it. Fifty years later, Stewart’s idea of what was and was not pornography had long since been abandoned as society changed its mind about what was permissible. However, for fibromyalgia, many physicians, including physician-experts, still hew to the idea of “I know it when I see it” [4].

What we now know for certain is that every set of published fibromyalgia criteria, beginning with Smythe and Moldofsky in 1976 [5], identified a different group of patients [6-11]. Even in 2021, two sets of expert research criteria had an unsatisfactory agreement, with one set identifying 73% more individuals with fibromyalgia in a general population study than the other [12]. In addition, general physicians’ knowledge about fibromyalgia and the de facto diagnostic agreement with published criteria was and is poor [13-15], even in specialty clinics [16]. In community practice, I know it when I see it is the rule.

What is fibromyalgia and how did it come about?

The substrate of fibromyalgia is musculoskeletal-related pain and non-pain symptoms. For those who first identified and developed the criteria for fibromyalgia, the disorder was carved from symptoms that, taken together, appeared excessive. By excessive physicians meant too much pain and too many symptoms or symptoms that are too severe, too emotionally ladened, or too unexplained. Excessiveness, severity, and causal understanding are judged in the eye of the medical *homme moyen sensuel*, observers whose ideas may change with knowledge or time or social pressure or location [17]. Some may carve from this symptom aggregate a specific group of symptoms to which names are attached such as fibromyalgia; others may see a bigger picture such as “functional somatic syndrome” or “bodily distress syndrome” or “central pain syndrome.” From this pool of symptoms, rules are applied and disorders are constituted. For fibromyalgia, the constituting of symptoms began in the 1930s. By a different logic, one could go back a century or forward by 30 or 40 years [18].

The development of fibromyalgia diagnosis

The early conceptualizers of fibromyalgia saw it as a musculoskeletal pain disorder that was frequently associated with “psychosomatic” factors and “life situations,” often disappearing when the “emotional stress” was over [19]. “It [pain and stiffness] may be widely scattered (generalized fibrositis) or be sharply localized” [20]; “Generalized aching and stiffness is the chief diagnostic symptom” [21]. The problem with fibromyalgia diagnosis was not just that the symptoms were qualitative and unmeasurable, but it was not clear just which symptoms (or physical findings) should be measured, nor how symptom severity should be accounted for. In addition, many physicians interpreted fibromyalgia symptoms and characteristics to be manifestations of a psychosomatic disorder or “psychogenic rheumatism” [22-24]. One report stated that “psychogenic rheumatism is encountered most frequently among middle-aged women. The typical complaint is of widespread pain and stiffness, often with report of swelling and paresthesia, but symptoms characteristically are vague” [22]. Reynolds warned that “labelling patients [as ‘fibrositis’] whose “symptoms stem from emotional disturbance may comfort physicians or patients by creating an aura of organicity about the psychogenic disorder.” Psychogenic rheumatism rapidly disappeared from the approved lexicon, but the concerns that psychological issues were central to fibromyalgia continued [25], bolstered to some extent by self-diagnosis inherent in fibromyalgia criteria and in the high rates of psychiatric illness among those diagnosed with fibromyalgia [26-28].

In 1977, Smythe and Moldofsky introduced the idea of “tender points” as a measure of decreased pain threshold, something they thought clearly separated fibromyalgia from other disorders [5]. Since 1981, with the introduction of the first widely used set of criteria, the Yunus criteria [6], and continuing through to the present, the following concepts contended in the criteria-based diagnosis of fibromyalgia: (1) the extent and location of musculoskeletal pain, (2) the presence or absence of “widespread pain,” (3) the presence or absence of non-musculoskeletal symptoms and their extent and severity, and (4) the measurement of tender points. It was not until 2010 and the American College of Rheumatology (ACR) 2010 preliminary criteria that measurable non-pain symptoms were added, as a four-item 0-12 symptoms severity score (SSS) [8]. With this addition, fibromyalgia criteria allowed a *de facto* case definition of fibromyalgia for the first time. By contrast, the 1990 ACR criteria said nothing about non-pain symptoms [7]; and the earlier Yunus criteria did not contain appropriate symptom variables, and could be satisfied by individuals with disparate symptoms and clinical findings [6]. The term ACR criteria is complex. For clarity and meaning, the 2010 preliminary criteria for fibromyalgia were ACR-endorsed criteria. Several years later, the ACR decided that they would no longer endorse “diagnostic criteria.” For that reason, the 2011 and 2011 modifications could not be submitted to the ACR for endorsement. We characterize the ACR 2011 and 2016 criteria as modifications of the original ACR 2010 criteria.

The most important departure from the fibromyalgia of the early conceptualizers was the requirement for symmetrical widespread pain and/or extensive pain that was first noted in the ACR 1990 criteria. Without this requirement, there was no way to reliably diagnose fibromyalgia as the non-pain symptoms were too many, too diffuse, and too difficult to measure. With the requirement of extensive pain implied by the widespread pain criterion, the idea of regional fibromyalgia came to an end. This loss was an important change because regional pain as part of fibromyalgia was inherent and sufficient in the ideas of the early conceptualizers [19-21]. Once extensive pain became a requirement, it was easy to introduce symptoms as additional criteria or diagnosis modifiers. The idea of less than widespread pain would emerge again with the development of the polysymptomatic distress scale and its use in regional, non-widespread pain disorders [29-31].

The key changes from the birth of fibromyalgia after the Second World War to the present followed a pattern of first requiring more extensive pain reports and later mandating increased symptoms scores [7-10]. The “AAPT Diagnostic Criteria for Fibromyalgia” that were proposed in 2020 called for pain in six of nine body areas [11], and the 2016 modification of the ACR criteria required pain in four of five body regions (widespread pain) and pain in at least seven of 19 specific pain sites [10]. The ACR’s 0-19 pain sites score constituted the widespread pain index (WPI). The 2010/2011 ACR criteria did allow non-widespread pain if there was also a very high SSS. However, the non-widespread pain allowance was removed in the 2016 criteria as it was found that it allowed people with limited regional pain syndromes to be (perhaps incorrectly?) identified as having fibromyalgia [32]. The tender point count of Smythe and Moldofsky [5], the central feature of the ACR 1990 criteria, was dropped for numerous reasons. These included the difficulty non-expert physicians had in performing the test; the recognition that the examination was unreliable and performed poorly; the knowledge that physicians and patients could influence the results; and the recognition that the tender point count was more likely measuring psychological distress than reduced pain threshold [33-36]. The lost promise of the tender point examination for diagnosis was the loss of an ostensibly “objective” test, as every other criteria-related measure of fibromyalgia was subjective and subject to potential manipulation by physicians and patients. As noted above, the 2010 through 2016 ACR-related criteria also required the presence of key non-pain symptoms of sufficient severity (SSS), in agreement with clinical observations of a general increase in the number and severity of symptoms [37]. From time to time, the possibility of a diagnosis of fibromyalgia based on laboratory or neurophysiologic abnormalities has been suggested. However, no valid scientific data have emerged to support such tools for clinical diagnosis. Now, in 2022, criteria for fibromyalgia that include self-reported pain and symptoms have wide but by no means universal [38-40] acceptance both in the clinic and in research studies. Fibromyalgia criteria have many purposes in addition to dichotomous diagnosis. Perhaps their most important purpose is to define fibromyalgia, deciding just how much severity is necessary for diagnosis, and which tools should be used. Fibromyalgia is not a natural kind and its diagnosis represents the opinion of a committee [41]. The fibromyalgia definition and its criteria may change in the future, as it has in the past.

Application of fibromyalgia criteria

If there are now generally agreed-upon criteria for fibromyalgia, it is fair to ask, how good are the diagnostic process and the criteria for fibromyalgia in the community where most of the diagnoses are made by primary care physicians, as well as in epidemiological studies where criteria application alone defines diagnosis. For fibromyalgia to be present according to the 2016 criteria, a patient must have (1) a WPI of ≥7 and an SSS of >5 OR a WPI of 4-6 and an SSS of ≥9; (2) Widespread pain, defined as pain in at least four of five regions; and (3) symptoms that have been generally present for at least three months. Moreover, there is no exclusion from fibromyalgia criteria for other diagnoses.

**Figure 1:**
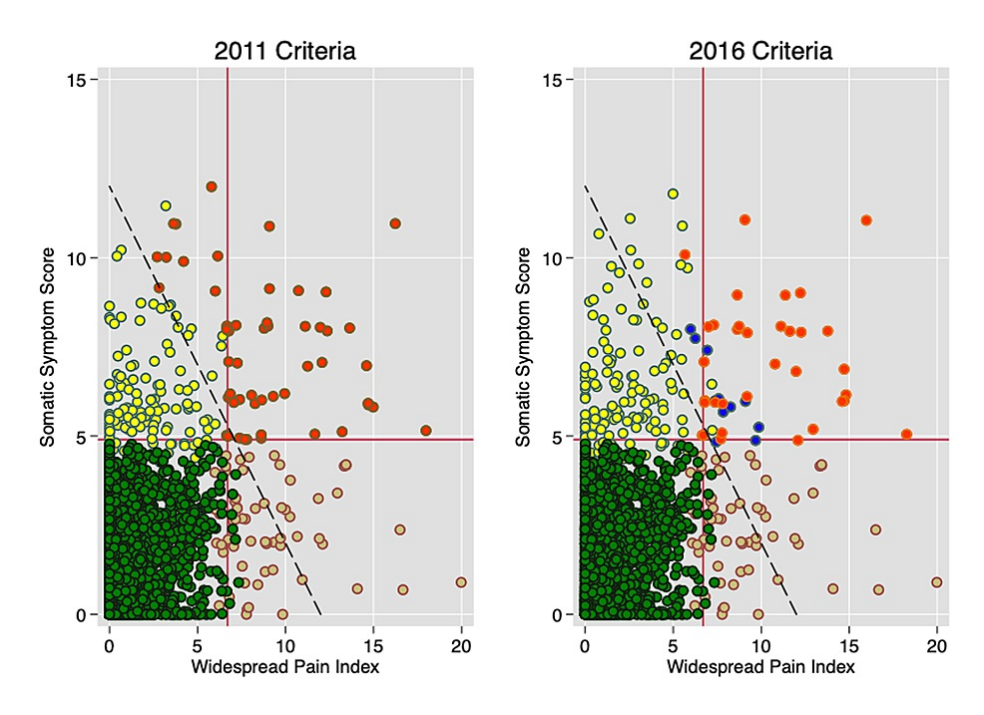
WPI and SSS scores in the German general population. The horizontal line at an SSS of 5 and the vertical line at a WPI of 7 represent fibromyalgia diagnostic level requirements. Red dots are fibromyalgia-positive cases. Blue dots are cases not qualifying because of the 2016 widespread pain requirement. Yellow dots are fibromyalgia-negative cases with high symptom scores. Tan dots are fibromyalgia-negative cases with high WPI scores. Green dots are fibromyalgia-negative cases with low symptom and WPI scores. The diagonal line represents a PSD value of 12 [1]. WPI: widespread pain index; SSS: symptom severity scale; PSD: polysymptomatic distress

Figure 1 shows the results of applying the modified ACR 2011 and 2016 criteria to a nearly perfectly executed population study in Germany in 2013 [1]. Red circles show fibromyalgia cases according to the 2011 (without widespread pain) and 2016 criteria (with widespread pain). The major approximate diagnostic (+/-) separation lines start at a WPI of 7 and an SSS of 5. The diagonal line identifies cases with polysymptomatic distress scale (PSD) scores of 12, greater PSD values are to the right of the line and lesser values to the left of the line. The PSD scale, also called the Fibromyalgia Severity (FS) scale, is a measure of the overall severity of the disorder, effectively the overall severity of fibromyalgia symptoms. It represents the sum of WPI and SSS scales, with a range of 0-31. Individuals with yellow circles have high levels of SSS but normal WPI scores; individuals with tan circles have the opposite, that is, high levels of WPI and normal levels of SSS. Individuals with blue circles have high levels of WPI and SSS but fail the criteria for other reasons, usually not satisfying the widespread pain requirements.

Application of fibromyalgia criteria in a primary care setting

**Figure 2:**
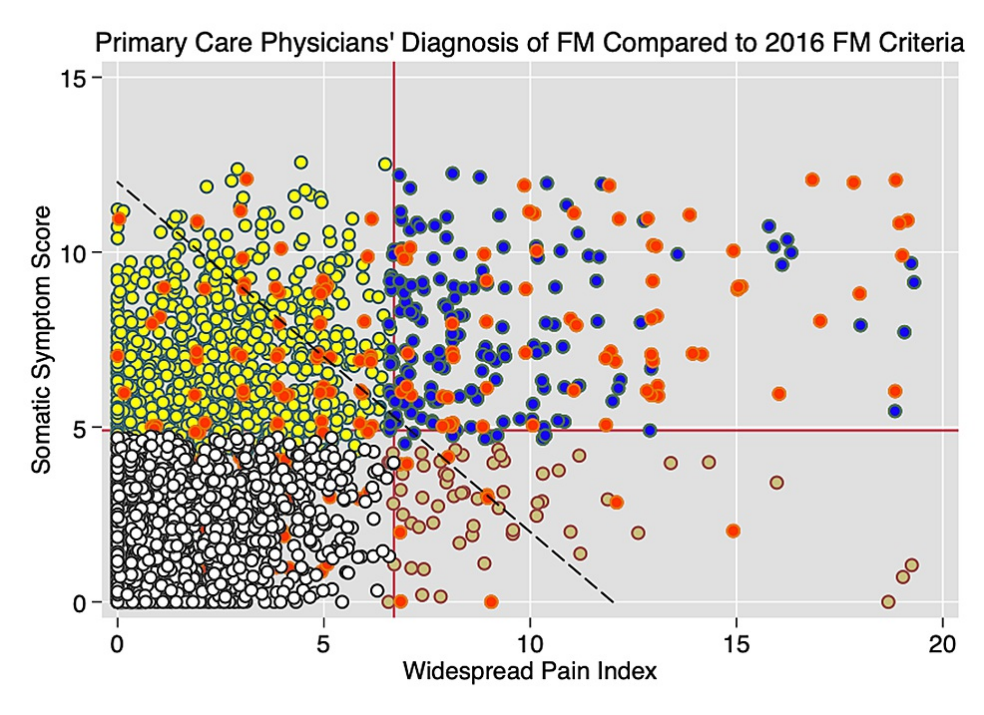
WPI and SSS scores in 3,276 US primary care patients. The horizontal line at SSS of 5 and the vertical line at WPI of 7 represent fibromyalgia diagnostic-level requirements. Red dots are fibromyalgia criteria-positive cases, as diagnosed by physicians. In the right upper quadrant, red dot cases satisfied the criteria. In the left upper quadrant, red dots cases did not satisfy the fibromyalgia criteria. Blue dots are fibromyalgia-positive cases that satisfy the criteria but were not diagnosed by physicians. Tan dots are fibromyalgia criteria and physician-negative cases with high WPI but low SSS. Yellow dots are fibromyalgia criteria and physician-negative cases with high SSS and low WPI. White dots are fibromyalgia criteria and physician-negative cases with low SSS and low WPI. Only cases in the right upper quadrant satisfy fibromyalgia 2016 criteria. The dashed diagonal line represents the line where the PSD score is 12 [2]. WPI: widespread pain index; SSS: symptom severity scale; PSD: polysymptomatic distress; FM: fibromyalgia

Figure 2 shows the classification of cases according to the 2016 criteria and physician diagnosis in 3,276 US primary care patients [2]. Correctly diagnosed criteria-based cases (determined from the fibromyalgia criteria questionnaire) appear in the right upper quadrant. Red circles indicate physician-diagnosed cases; other colors represent patients not diagnosed by physicians as having fibromyalgia. There are a very large number of patients who meet fibromyalgia criteria requirements but are not diagnosed with fibromyalgia (blue circles). There are also several patients diagnosed with fibromyalgia (red circles) who do not meet the criteria (red circles not in the right upper quadrant). The kappa values for the association of clinical diagnosis with criteria diagnosis is 0.296 (minimal agreement) and is 0.470 (moderate agreement) when criteria-positive; undiagnosed cases are excluded from the analysis. Red circled patients have substantially lower mean PSD severity scores than are found in criteria-based fibromyalgia, a finding based primarily on scores contributed by patients who do not satisfy criteria. The mean (standard deviation, SD) PSD for criteria-based German population (Figures 1, 2) satisfying criteria is 2016 criteria 17.3 (3.7), 2011 criteria 18.4 (3.4) compared with results from primary care physicians shown in Figure 2: 2016 criteria diagnosed fibromyalgia 18.4 (4.4), physician-diagnosed fibromyalgia (red dots): 12.4 (6.9). Of the 199 patients reporting a diagnosis of fibromyalgia in the primary care study of Figure 2, 65 or 35.4% met the 2016 criteria. These results of discordance between physician and criteria-based diagnosis are similar to those found using the US National Health Interview Survey (NHIS) [42]. Thus, based on many sources, physician-diagnosed fibromyalgia is considerably milder than the criteria-applied diagnosis. In fact, many individuals reporting a physician diagnosis of fibromyalgia in Figure 2 clearly do not have fibromyalgia, or at least do not satisfy the criteria. In addition, many individuals in the primary care setting who appear to satisfy fibromyalgia criteria have not received a diagnosis of fibromyalgia by clinicians. These findings have been replicated in a university rheumatology clinic [16]. Recently, 45% of patients referred to two pain rehabilitation centers in Denmark were found to have fibromyalgia, but only 19% had been diagnosed previously [43].

The difference between criteria and diagnosis based on clinical impression could reflect incorrect diagnosis or clinical improvement following correct diagnostic assignment or both. The loss of severity can also be seen graphically as many physician-diagnosed cases occur to the left of the diagonal in Figure 2. Also note that among those misdiagnosed, most have high symptom counts (left upper quadrant) but low WPI, indicating that physician diagnosis often gives strong weight to symptoms even without substantial pain. In this report from primary care practices, 64.8% of individuals studied are women, but among diagnosed cases of fibromyalgia, 84.0% are women. That percentage is much greater than the 59.6% of fibromyalgia-positive patients who are women seen in the German and other population studies [44]. Such data provide evidence of biased ascertainment and diagnosis favoring those with higher symptom scores and women.

Several other studies also support results that show incongruity between criteria and clinical diagnosis [42,45]. It is hard to overstate the importance of these results as fibromyalgia diagnosis can impact disability pensions, medical access, and approval of drugs by regulatory authorities, with personal and social consequences in many countries. If data such as these are correct, self-reported prevalence estimates, hospital records, and costs associated with fibromyalgia are also misinformed. In addition, popular usage takes on a reality of its own and the distinction between actual and possible is obscured. It would appear that there are at least two fibromyalgias, one the fibromyalgia of the medical research literature, and the other representing actual clinical practice.

How much “severity” is enough for fibromyalgia diagnosis?

The observation that levels of severity differ between clinical and criteria-based diagnosis raises the question of how the fibromyalgia severity level, the point where normal becomes abnormal, was determined, as there is no gold standard for fibromyalgia diagnosis. The first effort to define that point based on data came from the ACR 1990 criteria study where 16 rheumatology physicians diagnosed 293 fibromyalgia and 265 non-fibromyalgia cases in their practices based on their beliefs about fibromyalgia and then provided clinical measurements in a study setting for analysis [7]. The second effort occurred as part of the 2010 study which used 829 rheumatologist-diagnosed consecutive fibromyalgia cases and non-cases from 46 physician practices and then assessed a series of questionnaire items and tender point scores, allowing identifications of the best dividing point.

The AAPT 2019 criteria study did not primarily use clinical data in developing criteria. Instead, it used the ACR 1990 widespread pain definition and population-based surveys of widespread pain to standardize their pain site requirement [11]. The prevalence of fibromyalgia in the general populations was 73% greater with the AAPT criteria than with the 2016 modified ACR criteria, and the AAPT criteria selected individuals with less symptom severity and fewer pain sites [12]. Another criteria that defined fibromyalgia a priori was the 1981 Yunus criteria. To have fibromyalgia in that study patients had to have “symptoms of either generalized aching or stiffness, involving 3 or more areas” and “at least 4 well-defined tender points” out of 33 examined (approximately 12%) [6]. By contrast, the 1990 ACR criteria required 11/18 (61%) and approximately four or more areas of pain. The Yunus criteria were cited 1,277 times between 1981 and 1990. Although fibromyalgia in children may be very different from fibromyalgia in adults, the Yunus juvenile fibromyalgia criteria that used the same pain and tender point requirements are still in use (cited 398 times through 2021) [46]. Although it is hard to discern how much “easier” it would be to satisfy the Yunus adult criteria than the 2016 criteria, by using estimates of the minimum number of painful regions required to diagnose fibromyalgia in the 2016 criteria, we estimate that the Yunus criteria would have increased fibromyalgia prevalence by at least 50%.

Figures 1 and 2 provide further insight into the effect of varying key variables. Moving the vertical WPI line to the right or the left results in a greater or fewer number of patients satisfying criteria. Similarly, one can mandate more or less effect of non-pain variables by changing the level of the required symptom score. The presence or absence of the widespread pain requirement or its absence can further modify the cases selected. The system is logical, but rigid, with no simple method for handling exceptions. All criteria sets, when used in clinical care, however, allow clinicians the prerogative to take other factors into consideration in choosing to make or not make a diagnosis. However, criteria rules are not inviolable and can be altered. Consider, for example, committees that established the upper limits of blood pressure (or sodium, etc.) and then lowered the limits again and again [47,48], or of diseases or illnesses that have changed criteria or are no longer regarded as diseases [49,50].

The importance of polysymptomatic distress

The ACR associated 2010-2016 criteria used scores of the WPI and SSS to define fibromyalgia cases. Diagnosis alone, however, did not provide a useful measure of fibromyalgia severity as the range of symptom and outcome severity was large. When WPI and SSS are summed, however, the resultant scale provides a powerful and useful measure of severity that we have called the PSD scale, also called the FS scale [29]. Increasing levels of PSD are associated with every adverse outcome and scale, as well as with demographic and environmental predictors of fibromyalgia.

**Figure 3:**
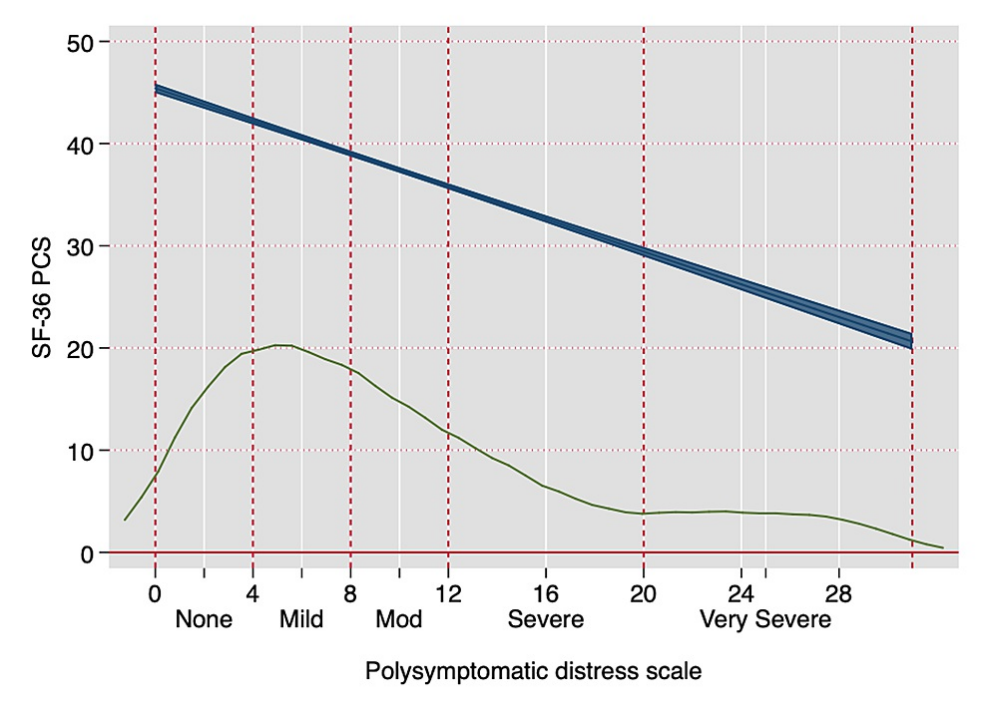
The relation between clinical severity as measured by the SF-36 PCS and the PSD scale in the NDB. The lower curve represents kernel density estimation of the PSD distribution. With increasing PSD values, which become less common, more abnormal PCS values are seen. The blue line represents the predicted values of SF-36 and 95% confidence intervals. SF-36: Short Form-36; PCS: Physical Component Score; PSD: polysymptomatic distress; NDB: National Data Bank for Rheumatic Diseases

**Figure 4:**
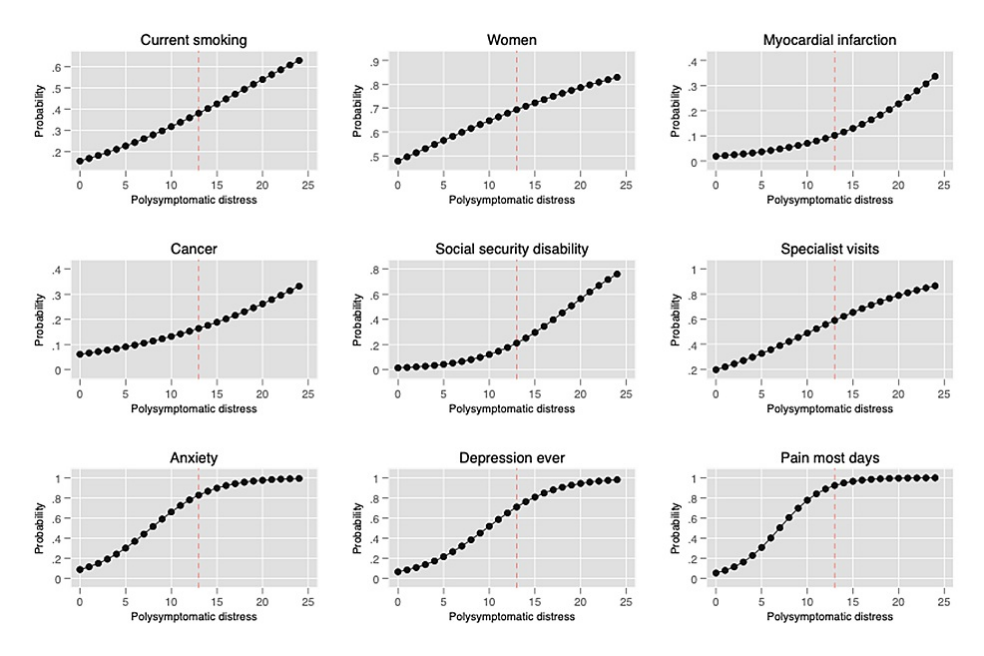
Association of PSD with selected status and outcomes in the National Health Interview Survey. PSD: polysymptomatic distress Reprinted after permission from PLoS One: Walitt B, Nahin RL, Katz RS, Bergman MJ, Wolfe F: The prevalence and characteristics of fibromyalgia in the 2012 National Health Interview Survey. PLoS One. 2015, 10:e0138024. 10.1371/journal.pone.0138024 [28].

**Figure 5:**
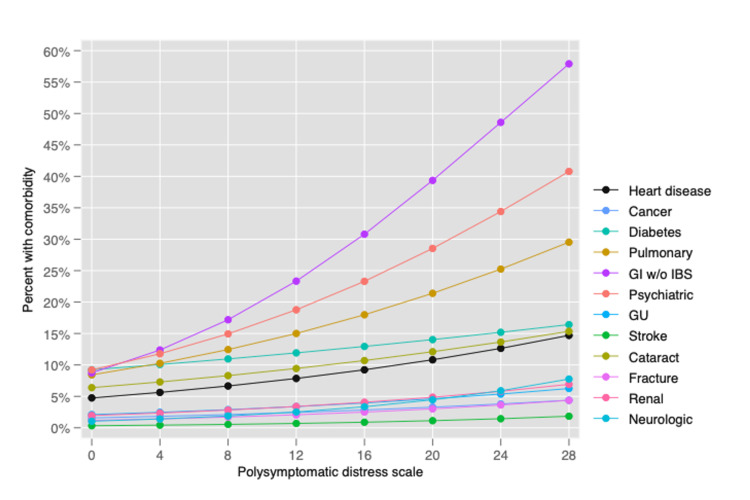
Association of PSD with a comorbid condition in patients with fibromyalgia in the NDB. Predicted percentage with comorbidity at levels of PSD. PSD: polysymptomatic distress; NDB: National Data Bank for Rheumatic Diseases; GU: genitourinary; GI: gastrointestinal; w/o IBS: without irritable bowel syndrome Modified from Wolfe F, Ablin J, Guymer EK, Littlejohn GO, Rasker JJ: The relation of physical comorbidity and multimorbidity to fibromyalgia, widespread pain, and fibromyalgia-related variables. J Rheumatol. 2020, 47:624-31. 10.3899/jrheum.190149 [52].

**Figure 6:**
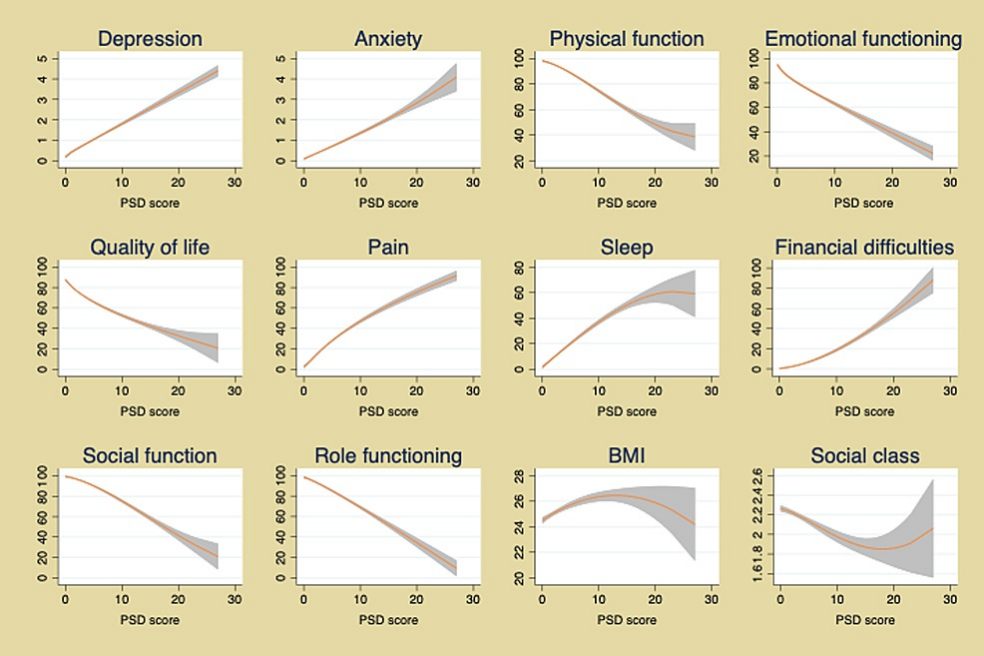
Association of PSD scores with clinical status and life events in the German population epidemiology study. Lines are predicted mean values and 95% confidence intervals. PSD: polysymptomatic distress; BMI: body mass index Modified from Wolfe F, Brähler E, Hinz A, Häuser W: Fibromyalgia prevalence, somatic symptom reporting, and the dimensionality of polysymptomatic distress: results from a survey of the general population. Arthritis Care Res (Hoboken). 2013, 65:777-85. 10.1002/acr.21931 [1].

The PSD scale also provides a simple method to assess improvement and worsening. PSD is a continuous scale, but to aid understanding of illness, categories of PSD have been suggested to identify clinically useful subgroups: (0-3, 4-7, 8-11, 12-19, 20-33), as shown in Figure 3 [3,51]. Figure 4 shows how the PSD can be used when applied to NHIS data [28]. Figure 5 demonstrates the relationship of PSD with the presence of various comorbid conditions [52], and Figure 6, from the German population data, shows its association with important clinical and life events [1]. In addition, the PSD is highly correlated with other measures of fibromyalgia activity and severity, such as the revised Fibromyalgia Impact Questionnaire (FIQR) and the modified Fibromyalgia Assessment Scale (FAS 2019mod), at r = 0.728 and r = 0.899, respectively [53].

The PSD scale provides important insights into the nature of fibromyalgia and fibromyalgia symptoms. Data from the above figures and many studies make it obvious that fibromyalgia is not a discrete disorder. It would be foolish, for example, to consider persons with PSD scores of 11 and 12 to really be different. A person with a PSD score of 11 but not a fibromyalgia diagnosis is much closer in severity to a person with a score of 12 (and a fibromyalgia diagnosis) than to a person with a score of 3. Such observations indicate the serious problems that arise from dichotomizing continuous scales [54]. In addition, persons who do not meet fibromyalgia criteria but have mild (4-7) and moderate (8-11) PSD scores have substantial symptomatic burdens and an increase in adverse outcomes, as shown in Figures 4-6.

However, there are more insights that can be drawn from the PSD components. WPI and SSS are correlated. In the primary care dataset, the correlation between WPI and SSS is 0.44, in the German population study 0.44, and in the NDB studies 0.53. In addition, this association is maintained in those with PSD scores of <12 (not fibromyalgia) as well as in those with scores of ≥12.

**Figure 7:**
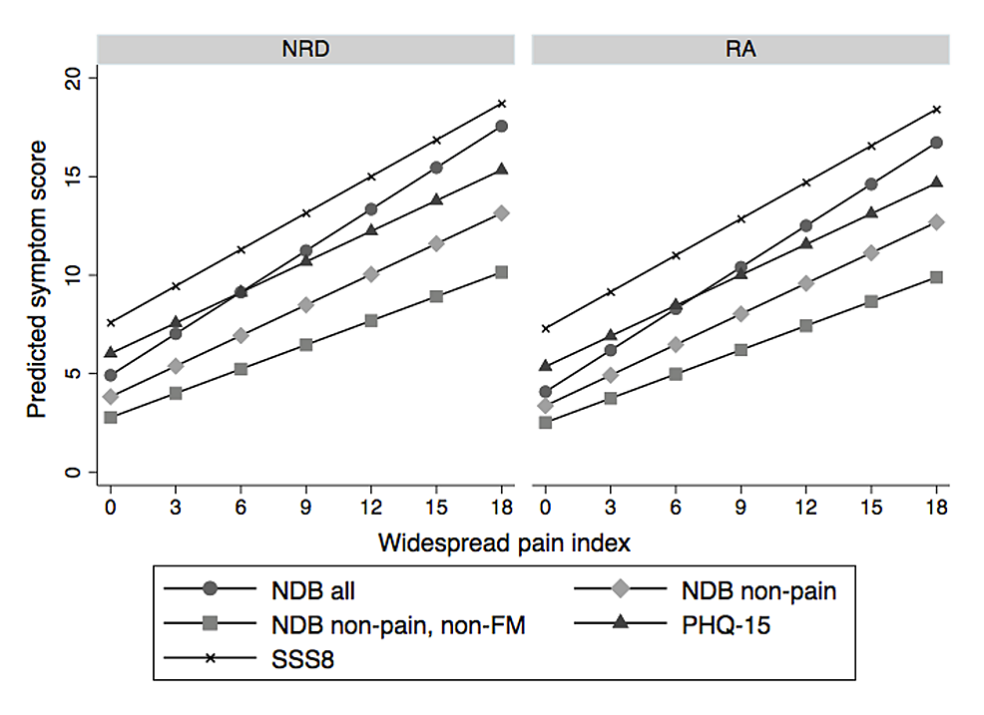
The relation between WPI and five symptom scales in the NDB. WPI: widespread pain index; NDB: National Data Bank for Rheumatic Diseases; NRD: patients with non-inflammatory disorders; RA: rheumatoid arthritis patients; NDB all: count of all somatic symptoms; NDB non-pain, non-FM: all symptoms that are not related to pain or fibromyalgia variables; NDB non-pain: symptoms not related to pain; SSS8: short symptom scale-8 [56]; PHQ-15: Patient Health Questionnaire-15 scale [55]

**Figure 8:**
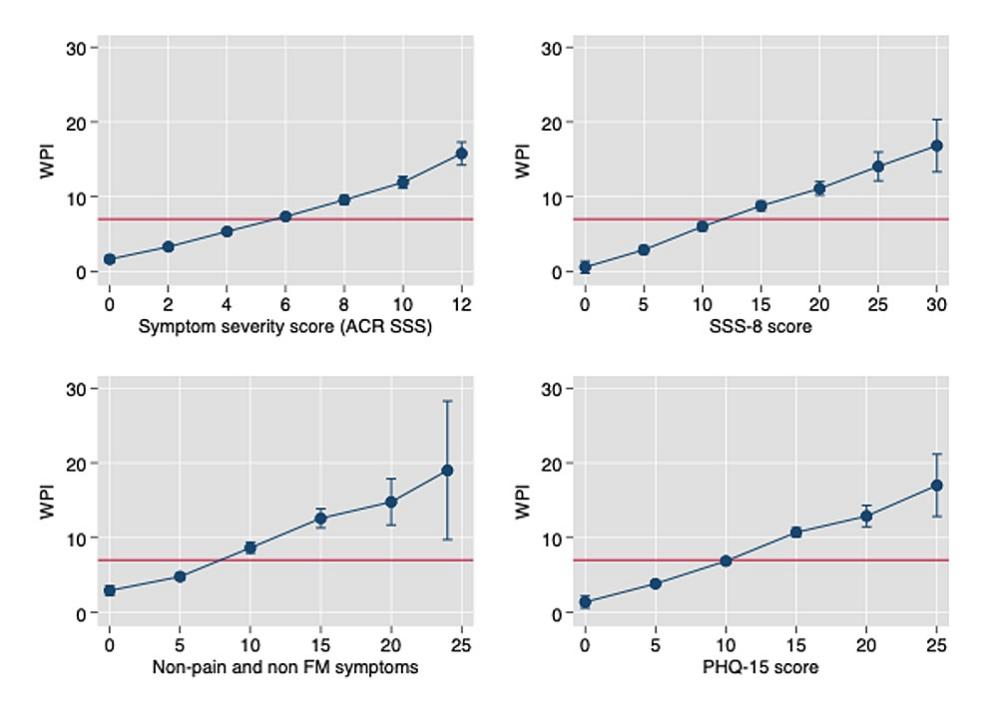
The relation between WPI and four symptom scales on the x-axis in NDB patients. The red horizontal line occurs at a WPI of 7, the level where high WPI scores begin [56]. WPI: widespread pain index; NDB: National Data Bank for Rheumatic Diseases; NRD: patients with non-inflammatory disorders; RA: rheumatoid arthritis patients; NDB all: count of all somatic symptoms; NDB non-pain, non-FM: all symptoms that are not related to pain or fibromyalgia variables; NDB non-pain: symptoms not related to pain; SSS8: short symptom scale-8 [57]; PHQ-15: Patient Health Questionnaire-15 scale

Figures 7, 8 show the linear relation between WPI and SSS [3]. Not only does SSS increase with increasing WPI but so do three other scales, the Patient Health Questionnaire-15 (PHQ-15) and SSS-8 somatic symptom scales, and a count of 24 non-pain, non-fibromyalgia symptoms [55,56]. The latter is an indication that WPI is associated with a general symptom increase, not just an increase in fibromyalgia-related variables. As a WPI score of ≥7 is a cut-off for a fibromyalgia diagnosis, the relationship between WPI and symptom variables exists below and above the fibromyalgia devising point.

What is wrong with fibromyalgia

The academic physician, Charles Engel, wrote in 2006 of fibromyalgia and similar disorders that “patients and clinicians gaze into the suffering and impact of their symptoms and see as many potential causes as there are symptom combinations. Whose explanation is best? When I am speaking with psychiatrists, the notion of somatoform symptoms goes largely unchallenged. When I am with rheumatologists, however, fibromyalgia is anything but a psychiatric problem and certainly not a somatoform disorder” [57]. The earlier concerns about symptoms related to “psychosomatic factors” and “psychogenic rheumatism” had been side-stepped by fibromyalgia criteria that were neutral to such concerns, in effect welcoming more persons into the fibromyalgia umbrella [19,22-24]. Despite present and past criteria and endorsement by various professional, governmental, and patient groups, most physicians have grave concerns about the nature and legitimacy of the disorder [25]. As an example, in a study of 524 internal medicine and rheumatology physicians, Goute reported that “more than 60% … would rather not have to care for the patient with fibromyalgia,” 96% felt that psychological factors were very important in fibromyalgia compared with 22% for lupus, and 37% thought that biologic factors were important or very important compared with 94% for lupus. Overall, 90% thought that symptom subjectivity was important or very important and 50% had important or very important suspicions about “secondary benefits.” When asked to rate the prestige (a measure of regard or esteem) of 38 different diseases in 1990, 2002, and 2014, a group of 291 Norwegian physicians ranked fibromyalgia the lowest, followed by anxiety, in each of the three periods [58].

The danger of psychogenicity to individuals with fibromyalgia and advocates for fibromyalgia was the widespread popular but wrong idea that “psychological” meant “not real.” Not only was the intellectual idea unacceptable but funding opportunities and sources would be strikingly different if fibromyalgia was perceived to be a psychological disorder. While psychosomatic medicine has strong support in many European countries, it has little support in the United States [59]. Consequently, academic physician advocates of fibromyalgia have increasingly framed fibromyalgia as primarily a pain disorder largely caused by neurobiological factors. In effect: “It’s real, it’s caused by biologic disease.” The literature for a primarily biological basis of fibromyalgia is very large, and the recently revised International Classification of Diseases 11th Revision classified fibromyalgia as a form of chronic widespread pain [60]. However, alternative perspectives of etiology place fibromyalgia in the category of bodily distress syndromes and functional somatic disorders [61]. Fink et al. indicated that “Bodily Distress Disorder … may unite many of the functional somatic syndromes and some somatoform disorder diagnoses. Bodily distress may be triggered by stress rather than being distinct diseases of non-cerebral pathology” [62]; and a pain-centered view of the world is too narrow [63]. Clauw et al. summarized an expert consensus pane: “In the final analysis, physical versus psychologic distinctions … might rest on preconception and perspective rather than a priori hypothesis testing. Whereas some observers have preferred to see a common physiological mechanism that explains symptoms, others have postulated common psychologic [features]” [64].

**Figure 9:**
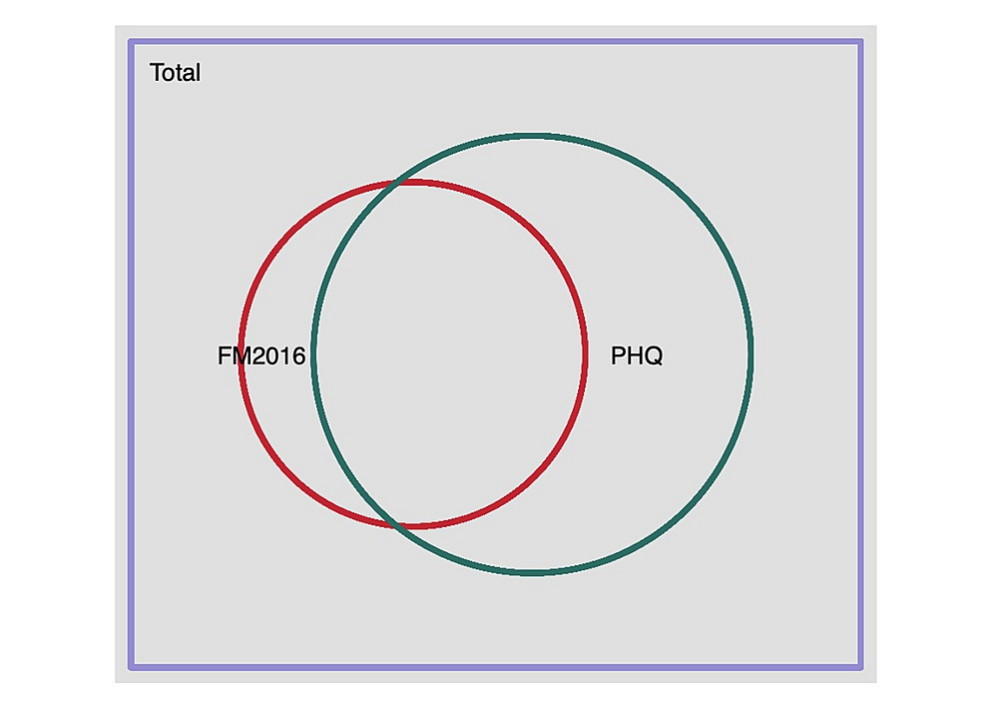
Venn diagram of FM 2016 and PHQ-15 at PHQ-15 of >9. N = 6,571 in the NDB. FM: fibromyalgia; PHQ-15: Patient Health Questionnaire-15 scale [55]; NDB: National Data Bank for Rheumatic Diseases

Figure 9, using NDB data from rheumatoid arthritis patients, shows the overlap between 2016 fibromyalgia criteria-positive patients and PHQ-15 subjects at levels considered medium or high (≥10) [3].

Among the 1,340 patients who were 2016 fibromyalgia positive, 78.1% had moderate or severe PHQ-15 scores. Data such as these that associate fibromyalgia with functional somatic or bodily distress syndromes are not merely suggesting that there are different names for the same thing [65]. Rather they suggest that there are different conceptual and etiological models. One model is primarily mechanistic and favors the role of central sensitization as a driving force of pain and consequent symptoms. The other falls into the category of “biopsychosocial,” in which illness is the complex interaction among biological, psychological, and social factors [66]. Highlighting that the distinction can be contentious, as an example, Fink et al. point out that, “… very active patient groups that aggressively spread misinformation on the social media. … [Can have] have some impact on research. The Institute of Medicine in the US report on CFS/ME avoids including any studies on psychological factors or treatment by stating that this is a medical condition and not a psychiatric one” [62].

Diagnosis: the remission/improvement problem

No set of criteria deals comprehensibly with how to approach fibromyalgia that remits or comes and goes or responds partially, though offhand suggestions exist [67]. To understand the life course of fibromyalgia requires following an unbiased selection of patients over substantial lengths of time. However, almost no studies have done that [68]. There are also no criteria for remission.

As noted above, many patients diagnosed by physicians do not meet fibromyalgia criteria when assessed with current criteria (Figure 3) [2,16,42]. In the primary care study [2], we were unable to find an association between time from diagnosis and failure to meet criteria. This suggests the possibility of a bad diagnosis rather than improvement, but it is possible that the results could be explained by patient improvement. Should such improved patients be considered to have fibromyalgia? Other diseases allow the diagnosis to be continued to be stated in the face of improvement, for example, in diabetes, hypertension, and seizure disorder. In addition, a special case exists if, on the initial patient-physician encounter, a patient has a high PSD, but not high enough to satisfy criteria. If that patient was improving, the diagnosis might be continued, but what if the patient had never reached the fibromyalgia criteria threshold?

The literature on improvement and remission is variable and incomplete. The life history of fibromyalgia patients reveals increased medical encounters and surgical events prior to a fibromyalgia diagnosis. Experienced clinicians note that many patients have “bouts” of fibromyalgia. Graham noted fibromyalgia disappearing when the “emotional stress” was over [19]. Goldenberg stated that “symptoms wax and wane [and] the state of fibromyalgia may come and go [40]. Masi and Yunus indicated that “limited clinical data suggest three basic patterns: remitting-intermittent; fluctuating-continuing; and progressive” [69]. In one study with careful follow-up, 24.3% were in remission two years after diagnosis [70]. One long-term (eight years) study of children with fibromyalgia indicated that the majority continued to suffer from pain and impairment in physical, social, and psychological domains [68].

There are numerous potential approaches to the improvement/remission problem. The fundamental problem is the inability, in patients who do not satisfy fibromyalgia criteria, to differentiate pain and non-pain symptoms that are not from fibromyalgia from pain and non-pain symptoms that are related to fibromyalgia. In the German general population, 68.0% of subjects have >0 and <12 PSD scores and 28.1% have PSD scores of >=4 and <12, the latter representing people with clinically important but non-fibromyalgia-level symptoms [42]. Some authors, for example, would require a PSD of <5 for remission [53], while some would use the same logic in regard to improvement and remission using other scales [67]. We believe that no fibromyalgia symptoms is not the same thing as no symptoms (i.e., zero symptoms). However, just looking at data it is not possible to tell, for example, sleep disturbance in fibromyalgia from sleep disturbance with other etiologies. One reason for this difficulty is that the symptoms that are important in fibromyalgia are universal symptoms.

If it is difficult to tell the meaning of apparent improvement, it is also difficult to attach fibromyalgia-appropriate meaning to subsyndromal levels of fibromyalgia variables. One possible solution to this conundrum is to think of fibromyalgia in terms of the spectrum of functional somatic syndromes and interpret all symptoms in that light. On a practical level, as a rule of thumb, approximately a 20% reduction from the PSD cut-off of 12 might be a substantial improvement, that is, a PSD value of 9-10 or less.

The problem of clinical measurement

If it is true, as it most certainly seems to be, that primary care physicians do not use formal criteria, it is even less likely that they would use tools such as the PSD from the fibromyalgia criteria questionnaire or the FIQR [71]. For physicians wanting simple tools, we noted that among NDB participants, PSD values correlate with PHQ-15 at 0.70 and with SSS-8 at 0.73. Either of these tools should be helpful [55,56]. However, the PSD questionnaire and the PHQ-15 are equally simple to complete; the PSD might be a better choice when the fibromyalgia diagnosis is used as it provides diagnosis information as well.

The problem of concomitant disease in the diagnosis of fibromyalgia

Criteria committees and clinicians have had considerable problems deciding how to consider patients for fibromyalgia diagnosis in the presence of concomitant diseases or nociceptive pain sites. The 1990 ACR criteria study specifically evaluated patients who were selected for study because they had “secondary or concomitant fibrositis.” Analysis of the data suggested criteria worked as well in “primary” fibromyalgia as in secondary/concomitant fibromyalgia and suggested the distinction between the two types be discarded [7]. NDB studies to revisit the issue with the 2010-2016 criteria came to the same conclusion [3]. Still, this issue was the subject of letters to the editor and long commentaries, particularly in the 2016 criteria paper [10].

Philosophically, why should back pain in a patient with anatomic radiographic changes be allowed as a pain site but not pain related to a joint impacted by rheumatoid arthritis? What is fibromyalgia, anyway? How can we really tell if a local area represents nociceptive or nociplastic pain or a mixture of both? Why cannot a patient with a disease such as rheumatoid arthritis also have fibromyalgia? What difference does it make? What if the pain is half nociplastic and half nociceptive? The 2016 criteria addressed the problem this way: “A diagnosis of fibromyalgia does not mean it is the patient’s only diagnosis or even the most important diagnosis. It is only an acknowledgement that the patient has symptoms of fibromyalgia and satisfies fibromyalgia criteria. It is for the clinician to decide the meaning and importance of the clinical findings. Finding that a patient satisfies fibromyalgia criteria is not, ipso facto, sufficient to define the entirety of the patient’s medical conditions” [10].

The problem of non-diagnosis

Figure 3 provides perspective on this issue as it can be seen that many patients are undiagnosed. A case can be made that diagnosing fibromyalgia is not helpful in most medical encounters, for example, in pneumonia or malignancy. On the other hand, the status of the variables that underlie fibromyalgia is always helpful to know even if no diagnostic name is appended to them. In a series of important studies of surgical outcomes, Brummett et al. have shown that fibromyalgia status and PSD predict worse outcomes [72]. The knowledge that a patient with rheumatoid arthritis satisfies fibromyalgia criteria can help with the management of rheumatoid arthritis even if no diagnosis of fibromyalgia is made. In addition, to diagnose fibromyalgia, physicians have to accept that the concept of fibromyalgia is the right approach; however, many physicians do not [73,74]. Some groups strongly resist the fibromyalgia idea. The German Association of General and Family Medicine dropped the German fibromyalgia guideline recommendations because they did not want to use the fibromyalgia label, and the German Pediatric Association insisted on using the code F45.41 (a special German code for a pain disorder with somatic and psychological factors) [75]. In the United Kingdom, psychiatrists summarized the views of many that fibromyalgia was “an unhelpful diagnosis for both patients and doctors” [76].

The problem of bad research

There are approximately 700 scientific articles published each year that are primarily about fibromyalgia. Most of these articles do not meet the standards for reporting and are biased in their selection of cases and controls [77]. For example, it is frequent to see descriptions such as “We studied x number of cases of fibromyalgia and normal controls.” With respect to fibromyalgia, it is essential to understand when and how the diagnosis of fibromyalgia was made and how the patients were recruited. For example, studies that use clinical or hospital records to identify fibromyalgia cases may be studying patients who no longer meet fibromyalgia criteria or never met fibromyalgia criteria [78]. In addition, it may well be that bothersome, complaining patients are given the diagnosis of fibromyalgia, while in reality others would meet the criteria, but fibromyalgia was not considered [36].

As noted above, the assertion that the patient has fibromyalgia is frequently wrong or if patients had fibromyalgia, they may not have it now. In addition, the spectrum of fibromyalgia severity is wide [53]. For example, fibromyalgia patients with a PSD of ≥20 have more severe fibromyalgia symptoms and worse medical outcomes than those with scores of <20, as shown in Figures 4-7, A second critical issue is the nature of the control or comparison group. Should they be subjects with PSD scores of 0-2, or should they be selected from the full range of subjects who do not satisfy fibromyalgia criteria? If investigators select the most normal of controls and then fibromyalgia patients with PSD scores of ≥20, almost any hypothesis being tested will be positive, including objective neurobiological studies.

Subjects are selected with bias. Compared with population studies where the female-to-male ratio of fibromyalgia cases is close to 1:1, in clinical studies, the female-to-male ratio may be as high as 8/9:1. This reflects (1) the tendency of women to seek medical care for fibromyalgia-like symptoms compared to men. Of equal importance (2), it represents the observation that physicians suspect fibromyalgia when women present with symptoms more than when men do. In addition, individuals with psychological issues are more likely to be candidates for the fibromyalgia diagnosis than those without such symptoms. These biases occur at the primary care level and in the referral process. Aronowitz considers that what might be thought of as bias actually represent framing [79]. That is, rather than considering sex and psychological status as part of a bias, diagnosis is socially constructed so that sex and psychological status become part of an unspoken characteristic of the disorder. Note in Figure 3 that among fibromyalgia diagnosed patients who do not meet criteria, many more have high symptoms scores, but low WPI scores. Issues such as this should be clearly described in the methods sections of studies but are usually not. An additional important problem is the inadequate descriptions of study methods and details. Between 2007, when the STROBE (STrengthening the Reporting of OBservational studies in Epidemiology) checklist of observational study recommendations was published [77], and 2021, Google Scholar reported 9,320 unique articles with fibromyalgia in their title. The STROBE checklist was cited 21 times in that group of fibromyalgia references. The checklist makes recommendations regarding descriptions that are of profound interest to fibromyalgia research.

Fibromyalgia is a nociplastic pain problem

Central sensitization or nociplastic pain is a critical process in fibromyalgia [80]. However, as the central driving mechanism for fibromyalgia, it may be incorrect, or at least reflect an incomplete model. Data from the NDB, primary care, and German population study show that increases in the number of pain sites, as measured by the WPI, are associated with increases in the SSS (or vice versa) across the entire spectrum of PSD (Figures 7, 8). In the primary care dataset, the correlation between SSS and WPI is 0.44, in the German population study 0.44, and in the NDB 0.53. In addition, this association is maintained in those with PSD scores of <12 (not fibromyalgia) as well as those with scores of ≥12 (fibromyalgia (+)). If symptom increase is related to pain, it will also be related to pain at levels less than those that define fibromyalgia. In addition, not all symptoms are the result of central nervous system dysregulation. For example, patients may have fatigue or difficulty with sleep from nociceptive pain whether or not they have fibromyalgia. The full role of central sensitization and its relation to fibromyalgia and non-fibromyalgia remains to be elucidated.

## Conclusions

In the 45 years since it was first named fibromyalgia, it has taken on generally accepted criteria. Much is now known about the pain of fibromyalgia, including nociplastic pain and central sensitization. Serious, well-performed scientific studies have established beyond doubt that fibromyalgia pain and distress are “real.” Similarly, evidence of the importance of psychosocial symptoms has accumulated, and psychologic features are accepted as part of the disorder. Despite these findings, fibromyalgia has only partial acceptance, and diagnosis is uncertain and unpredictable. Factions contend as to whether fibromyalgia should be best considered a primary pain disorder fueled by central sensitization or a more broadly based functional somatic syndrome or bodily distress disorder. In addition, features of fibromyalgia exist at subsyndromal levels in almost all medical disorders in which there is pain, suggesting that pain and accompanied distress found in fibromyalgia might be a universal condition of mankind at differing levels of severity.
